# Otitis Media and Relevant Clinical Issues

**DOI:** 10.1155/2012/720363

**Published:** 2012-06-10

**Authors:** Jizhen Lin, Joseph E. Kerschner, Per Cayé-Thomasen, Tetsuya Tono, Quan-An Zhang

**Affiliations:** ^1^Department of Otolaryngology, Head and Neck Surgery, School of Medicine, University of Minneosta, Minneapolis, MN 55455, USA; ^2^Department of Otolaryngology & Communication Sciences, Medical College of Wisconsin, Milwaukee, WI 53226, USA; ^3^Department of Oto-Rhino-Laryngology, Head and Neck Surgery, Gentofte University Hospital of Copenhagen, 2900 Hellerup, Denmark; ^4^Department of Otorhinolaryngology, Head and Neck Surgery, University of Miyazaki, Miyazaki 889-1692, Japan; ^5^Department of Otolaryngology, Head and Neck Surgery, Second Clinical Medical College, Xi'An Medical University, Xi'An 710049, China

Otitis Media (OM), an infection in the space behind the ear drum, is one of the most common childhood infectious diseases worldwide. More than 70% of all children have at least one middle ear infection by the age of six years. Among them, approximately 5–10% of patients turn to chronic OM which eventually causes hearing loss and sequelae. 

The pathogenesis of chronic and/or recurrent OM are multifactorial, including genetic predisposition, dysfunction of the Eustachian tube, impairment of the mucociliary system, hyperproduction of mucins, metaplasia/hyperplasia of mucous cells, and active secretion of ions and water. No matter what contributes to the development of the disease, mucin production and mucous cell metaplasia/hyperplasia are always problems in this disease. 

Mucous cell metaplasia/hyperplasia occur in the middle ear mucosa and tend to drive OM into a chronic status, leading to abundant production and accumulation of mucus in the middle ear cavity. Under the circumstances, this leads to the development of an OM entity, that is, OM with mucoid effusion. Mucous cell metaplasia/hyperplasia is frequently associated with chronic purulent otorrhea when the tympanic membrane is performed, making OM irreversible and intractable. However, it is unclear how this event initiates at an acute setting of OM and deteriorates in the chronic stage of OM. 

In this particular issue, there are seven papers in total, covering the clinical areas and basic sciences relevant to OM. In the clinical areas, there are five papers, dealing with application of auditory brainstem response in the operating room, measuring thickness of middle ear mucosa with MRI and CT, hearing loss in cleft palate patients, otoacoustic emissions in children with OM with effusion, and appraisal of pneumococcal vaccine trials. In the basic sciences, there are two review articles, focusing on the role of Atoh1 in mucous cell metaplasia and mucin production and mucous cell metaplasia/hyperplasia, respectively. 

In these two review articles, what has been discussed includes the cellular events through which mucous cells are induced from general epithelial stem cells; bacterial metabolites/inflammatory cytokines that modulate the development of mucous cells in the middle ear, transcription factors and other molecules that are responsible for differentiation of mucous cells in the middle ear, possible factors that increase the risks for mucous cell metaplasia/hyperplasia, and signal pathways that are involved in the mucin disorders and mucous cell metaplasia/hyperplasia.

*Jizhen Lin* *Jizhen Lin* *Joseph E. Kerschner* *Joseph E. Kerschner* *Per Cayé-Thomasen* *Per Cayé-Thomasen* *Tetsuya Tono* *Tetsuya Tono* *Quan-An Zhang* *Quan-An Zhang*

